# Mena^calc^, a quantitative method of metastasis assessment, as a prognostic marker for axillary node-negative breast cancer

**DOI:** 10.1186/s12885-015-1468-6

**Published:** 2015-06-27

**Authors:** Catherine L. Forse, Seema Agarwal, Dushanthi Pinnaduwage, Frank Gertler, John S. Condeelis, Juan Lin, Xiaonan Xue, Kimberly Johung, Anna Marie Mulligan, Thomas E. Rohan, Shelley B. Bull, Irene L. Andrulis

**Affiliations:** 1 Department of Laboratory Medicine and Pathobiology, University of Toronto, Toronto, ON Canada; 2 Department of Pathology, Yale University School of Medicine, New Haven, CT USA; 3 Department of Pathology, Georgetown University, Washington, DC USA; 4 Lunenfeld-Tanenbaum Research Institute, Mount Sinai Hospital, Toronto, ON Canada; 5 Department of Biology and Koch Institute for Integrative Cancer Research, Massachusetts Institute of Technology, Massachusetts, MA USA; 6 Department of Anatomy and Structural Biology, Gruss Lupper Biophotonics Center, Integrated Imaging Program, Albert Einstein College of Medicine, New York, NY USA; 7 Department of Epidemiology and Population Health, Albert Einstein College of Medicine, New York, NY USA; 8 Department of Therapeutic Radiology, Yale University School of Medicine, New Haven, CT USA; 9 Laboratory Medicine Program, University Health Network, Toronto, ON Canada; 10 Dalla Lana School of Public Health, University of Toronto, Toronto, ON Canada; 11 Department of Molecular Genetics, University of Toronto, Toronto, ON Canada; 12 Department of Pathology & Laboratory Medicine, Mount Sinai Hospital, Toronto, ON Canada

**Keywords:** Mena, Metastasis, Breast cancer, Axillary node negative, Prognostic marker

## Abstract

**Background:**

Mena^calc^ is an immunofluorescence-based, quantitative method in which expression of the non-invasive Mena protein isoform (Mena11a) is subtracted from total Mena protein expression. Previous work has found a significant positive association between Mena^calc^ and risk of death from breast cancer. Our goal was to determine if Mena^calc^ could be used as an independent prognostic marker for axillary node-negative (ANN) breast cancer.

**Methods:**

Analysis of the association of Mena^calc^ with overall survival (death from any cause) was performed for 403 ANN tumors using Kaplan Meier survival curves and the univariate Cox proportional hazards (PH) model with the log-rank or the likelihood ratio test. Cox PH models were used to estimate hazard ratios (HRs) for the association of Mena^calc^ with risk of death after adjustment for HER2 status and clinicopathological tumor features.

**Results:**

High Mena^calc^ was associated with increased risk of death from any cause (*P* = 0.0199, HR (CI) = 2.18 (1.19, 4.00)). A similarly elevated risk of death was found in the subset of the Mena^calc^ cohort which did not receive hormone or chemotherapy (n = 142) (*P* = 0.0052, HR (CI) = 3.80 (1.58, 9.97)). There was a trend toward increased risk of death with relatively high Mena^calc^ in the HER2, basal and luminal molecular subtypes.

**Conclusions:**

Mena^calc^ may serve as an independent prognostic biomarker for the ANN breast cancer patient population.

**Electronic supplementary material:**

The online version of this article (doi:10.1186/s12885-015-1468-6) contains supplementary material, which is available to authorized users.

## Background

The majority of women diagnosed with axillary node-negative (ANN) breast cancer have a good prognosis; however, approximately 20 % of patients will experience a recurrence and die from systemic disease. Studies suggest that the risk of recurrence may depend on biologic subtype [1–3]. Gene expression and immunohistochemical marker profiling have been used to divide breast cancers into subtypes (i.e., luminal, basal-like, human epidermal growth factor 2 (HER-2) positive) which differ in terms of prevalence, recurrence risk, and sensitivity to chemotherapy [4–6]. The identification of prognostic markers for ANN breast cancer in order to detect patients who would receive the most benefit from adjuvant systemic therapy would improve survival and decrease the number of patients exposed to unnecessary treatment.

Mena is a pro-motility protein that is a member of the Enabled (Ena)/vasodilator-stimulated phosphoprotein (VASP) family of actin polymerization regulators [7]. It controls the geometry of assembling F-actin networks by antagonizing the activity of capping proteins at elongating actin filaments [8]. The protein is overexpressed in primary and metastatic breast cancers [9, 10], is particularly over-expressed in migratory and disseminating subpopulations of tumor cells *in vivo* [11], and has been shown to have an important role in breast cancer metastasis in both *in vitro* and *in vivo* experimental models [12]. It is an essential member of the Invasion Signature, a collection of transiently expressed proteins that control chemotactic and migratory behavior in primary rat, mouse and human mammary tumors [13–16]. In mouse models of breast cancer, forced overexpression of Mena increased lung metastases [17–20], while Mena deficiency decreased tumor burden by delaying tumor invasion, intravasation and dissemination to the lungs [20].

Studies of breast cancer cell migration and dissemination during metastasis at single cell resolution using multiphoton imaging in both mouse and human mammary tumors have led to the identification of microanatomic sites called Tumor MicroEnvironment of Metastasis (TMEM) [18, 19, 21, 22]. TMEM are sites of localized vascular permeability induced by macrophage vascular endothelial growth factor (VEGF) release where tumor cells intravasate [22].

Tumor cell migration toward TMEM *in vivo* occurs in association with macrophages and involves epidermal growth factor (EGF)/colony stimulating factor 1 (CSF-1) paracrine signaling [18, 23]. Studies of these migratory tumor cells led to the identification of the Invasion Signature which contains pathways up-regulated in gene expression and/or protein activity in tumor cells, with migration and TMEM assembly activity leading to transendothelial migration at TMEM and dissemination to distant sites [13, 16, 19, 24]. These pathways involve epithelial-to-mesenchymal transition-associated differential expression of Mena isoforms [18, 19].

Mena is alternatively spliced into multiple isoforms with Mena^INV^ and Mena11a being the best characterized in breast cancer [11, 17–19]. Mena^INV^, an invasive isoform, is spontaneously over-expressed in the migratory and disseminating subpopulation of tumor cells in primary mammary tumors of rat, mouse and humans [11, 16]. It confers a potent pro-invasion, pro-metastatic phenotype when expressed in breast cancer cells by potentiating their chemotactic invasion/migration response to EGF and by promoting discohesive cell motility [11, 17–19]. Mena11a, which contains a 21 amino acid exon insertion, is down-regulated in invasive breast tumor cells [11] and is down-regulated when human mammary epithelial cells undergo epithelial-to-mesenchymal transition [25]. Mena11a expression in breast cancer cells causes formation of a poorly metastatic tumor which does not respond to EGF signaling *in vivo* [18]. Furthermore, tumor cells with elevated transendothelial migration activity, isolated from breast cancer patients by fine needle aspiration, have spontaneously elevated Mena^INV^ and suppressed Mena 11a expression [26]. In addition, patients with elevated Mena^INV^ and decreased Mena 11a expression have greatly elevated TMEM counts [26].

Mena has shown promise as a prognostic marker for breast cancer. Its expression as a component of TMEM is associated with an increased risk of distant metastases in breast cancer patients [27, 28].

Mena expression in Mena^calc^, a multiplexed quantitative immunofluorescence-based method which takes into consideration the differential expression of Mena protein isoforms, is also associated with poor outcome [29]. Mena^calc^ involves subtracting the protein expression of the non-metastatic Mena11a isoform from total Mena expression in tumors to give an estimate of the invasive Mena isoforms. In two tumor cohorts unselected for nodal status, Mena^calc^ was associated with decreased disease-specific survival independent of patient age, receptor status and tumor size [29]. While Mena^calc^ was prognostic for poor outcome in node-positive patients, its role as a prognostic marker for ANN patients was unclear.

In this report, we evaluate the prognostic value of Mena^calc^ in a cohort of ANN patients. Our primary objective was to determine if there was an association between Mena^calc^ and overall patient mortality. A secondary objective was to determine if there was an association between Mena^calc^ and mortality within subgroups defined by (1) adjuvant treatment received, and (2) breast cancer molecular subtypes. These associations could help to identify patient populations more likely to benefit from Mena^calc^ testing.

## Methods

### Patient cohort and clinical follow-up

The characteristics of a prospectively ascertained consecutive series of 1561 ANN cases enrolled from eight Toronto hospitals between September 1987 and March 1993 and clinically followed for recurrence and death have been described previously [30, 31]. In brief, all women who had ANN invasive breast cancer pathologically confirmed at the participating centers were potentially eligible. The pathology report was used to determine the initial eligibility (which required clear resection margins and at least four lymph nodes sampled), pathologic size of the invasive component (centrally reviewed at Mount Sinai Hospital, Toronto, ON, Canada), presence of vascular or lymphatic invasion by tumor cells, estrogen receptor (ER) status, progesterone receptor (PgR) status, nuclear grading, histologic grading, and histologic subtype of the invasive and intraductal components (if present). Imaging (bone scan and abdominal ultrasound or abdominal computed tomography scan) and chest x-ray were required for patients with T2 tumors. If the patient was eligible on the basis of pathology, staging and age (between 18 and 75 years inclusive), the surgeon invited the patient to participate and provided a signed consent form. Patients were excluded from recruitment into this ANN cohort if (1) No tumor specimen was provided for analysis, (2) no axillary dissection was performed as part of surgical management, (3) less than four lymph nodes were biopsied and analyzed, (4) pathology revealed that the patient was diagnosed with carcinoma-in-situ disease (i.e., no invasive component), (5) the patient had distant metastases at the time of diagnosis, (6) the patient had synchronous primary breast tumors, (7) the patient had a previous breast malignancy, and (8) the patient had a secondary malignancy other than non-melanoma of the skin and carcinoma-in-situ of the cervix. Charts were reviewed every 3 months in the first 2 years after diagnosis, every 6 months until 5 years after diagnosis, and annually thereafter. Patient status on July 10, 2002 was used to determine survival times and censoring status using clinical follow-up data.

Approval of the study protocol was obtained from the research ethics boards of Mount Sinai Hospital (#01-0313-U) and the University Health Network (#02-0881-C), Toronto. Written-informed consent was received from all study participants. In the preparation of this paper, we used the reporting recommendations for tumor marker prognostic studies (REMARK) to present our results [32].

### TMA construction and IHC staining

Tissue microarrays (TMAs) were constructed from tumors of 888 women and biomarker status was determined as described below. Areas of invasive carcinoma were selected from a hematoxylin and eosin-stained section of each tumor and two 0.6-mm cores of tissue were taken from the corresponding areas of the paraffin block. The selected donor cores were embedded in a paraffin block and 4-μm sections were cut from this recipient block and used in series for immunohistochemical (IHC) staining. Microwave antigen retrieval was carried out in a Micromed T/T Mega Microwave Processing Lab Station (ESBE Scientific). Slides were pretreated at 115 °C for 12 minutes in TTMega Tris (pH 9.0) and incubated with antibodies to ER (clone 6 F11, 1:75 dilution, Vector, Burlington, ON, Canada), PgR (clone PgR1294, 1:1000 dilution, DAKO, Glostrup, Denmark), p53 (clone D.07, 1:400 dilution, ID Lab), or CK5 (clone XM26, 1:400 dilution, Vector, Burlington, ON, Canada). Alternatively, slides were pretreated with pepsin at 37 °C for 10 minutes and then incubated with antibodies to EGFR (clone 31G7, 1:25 dilution, Zymed, South San Francisco, CA, U.S.A.) or HER-2 (clone CB11/TAB250 (cocktail), 1:300 dilution, Novocastra, Newcastle upon Tyne, U.K. and Zymed, South San Francisco, CA, U.S.A.). Sections were developed with diaminobenzidine tetrahydrochloride and counterstained in Mayer’s hematoxylin.

### Antibodies and multiplexed immunofluorescence staining for Mena

The TMAs were deparaffinized by melting at 60 °C in an oven equipped with a fan for 20 minutes followed by 2x xylene treatment for 20 minutes. Slides were then rehydrated and antigen retrieval was done in citrate buffer (pH 6.0) at 97 °C for 20 minutes in a PT module (Labvision, Kalamazoo, MI, U.S.A.). Endogenous peroxidase was blocked by using 0.3 % hydrogen peroxide in methanol followed by incubation of slides in a blocking buffer (0.3 % bovine serum albumin in TBST (0.1 mol/L of TRIS-buffered saline (pH 7.0) containing 0.05 % Tween-20)) for 30 minutes at room temperature. Slides were incubated with a cocktail of mouse anti-pan-Mena (1:1000 dilution, BD Biosciences, San Jose, CA, U.S.A., catalog number 610693) mixed with rabbit anti-Mena11a (1:500 dilution of 1 mg/ml stock, generated in the lab of FG) in the blocking buffer overnight at 4 °C. After washing away the primary antibodies, slides were incubated with secondary antibody (goat anti-rabbit conjugated to horseradish peroxidase, Jackson ImmunoResearch Laboratories Inc., West Grove, PA, U.S.A.) to target Mena11a for one hour. After washing, slides were incubated with biotinylated tyramide (Perkin Elmer, Waltham, MA, U.S.A.) diluted at 1:50 in amplification buffer for 10 minutes. After washing, peroxidase activity was quenched by 2x treatment with benzoic hydrazide (100 mM in PBS) with 50 mM hydrogen peroxide for seven minutes each. After washing, slides were incubated for an hour with goat anti-mouse envision (DAKO, Carpinteria, CA, U.S.A.) followed by treatment with a chicken anti-Pan cytokeratin (1:100 dilution, generated in house) for 2 hours at room temperature. Slides were washed and then incubated with goat anti-chicken conjugated to Alexa546 (Invitrogen, Grand Island, NY, U.S.A.) to visualize cytokeratin and streptavidin conjugated to CY7 (750 nm, Invitrogen, Grand Island, NY, U.S.A.) to visualize Mena11a for an hour. After washing, slides were treated with CY5 conjugated tyramide (1:50 dilution; Perkin Elmer, Waltham, MA, U.S.A.) in amplification buffer for 10 minutes to visualize pan-Mena. Slides were mounted with ProLong gold mixed with DAPI (Molecular Probes, Grand Island, NY, U.S.A.). Serial sections of the index array used for assay standardization [33] were stained alongside each cohort to assess the assay reproducibility. An additional serial section of the index array was stained with each experiment with no primary antibodies as a negative control.

Of the 888 tumors submitted for Mena multiplex immunofluorescence staining, 403 had sufficient tumor material to permit reliable interpretation of pan-Mena and Mena11a.

### Automated quantitative analysis (AQUA) and calculation of Mena^calc^

The automated quantitative analysis (AQUA) technology (HistoRx, Branford, CT, U.S.A.) allows quantitative measurement of biomolecules in subcellular compartments as described previously [34, 35]. Briefly, a series of monochromatic images for each histospot was captured using a PM-2000 microscope (HistoRx, Branford, CT, U.S.A.) equipped with an automated stage. A binary ‘tumor mask’ was created using cytokeratin staining of the histospot representing only epithelial cells and excluding stromal features. AQUA scores for both pan-Mena and Mena11a were calculated by dividing the signal intensity by the area of the specific compartment (in this case within the tumor mask area). Normalized AQUA scores for both targets (pan-Mena and Mena11a) were used to calculate the Mena^calc^ fraction for each histospot by subtracting the z score of Mena11a from the z score of pan-Mena as described previously [29]. At the end of this procedure, Mena^calc^ was obtained for 403 tumors from the ANN cohort.

### Subgroup definitions

#### Treatment subgroups

The study group (n = 403) was divided into 2 groups based on adjuvant treatment: those who received no systemic adjuvant treatment and those who received any systemic adjuvant treatment (hormonal and/or chemotherapy). The two groups were called untreated (n = 142) and treated (n = 261) respectively.

#### Molecular subtypes

The tumors were divided into molecular subtypes using IHC-TMA markers described in previous publications [36–38]. Tumors positive for HER2 protein overexpression, regardless of ER status, were assigned to the HER2 subtype. Tumors negative for HER2 and ER and positive for one or both of CK5 and EGFR were assigned to the basal subtype. Tumors negative for HER2 but positive for ER were assigned to the luminal subtype, regardless of CK5 status.

### Statistical analysis

Pearson’s correlation coefficient (*R*) was used to assess the reproducibility of the multiplexed assay between near-serial sections of the index assay as described previously [29]. Pan-Mena and Mena11a AQUA scores, and Mena^calc^ values from two independent cores for each histospot were averaged and the averages were used for final analysis. Mena^calc^ scores were categorized as scores that were at or above the median (Mena^calc^ high) or below the median (Mena^calc^ low). This median cutoff was selected because of the division noted in the Kaplan Meier (K-M) survival curves generated from quartile groups. The chi-square test or Fisher’s exact test was used to analyze the Mena^calc^ marker associations with clinical-pathological tumor variables. Analysis of the association of overall survival (OS) with marker status was performed using K-M survival curves and the univariate Cox proportional hazards (PH) model with the log-rank or the likelihood ratio test. Multivariate analyses by the Cox PH model were conducted to assess the contribution of Mena^calc^, in addition to HER2 status (using IHC data), hormone receptors (using IHC data) and other clinical-pathological tumor variables. Hazard ratios (HRs) and 95 % confidence intervals (CI) were also estimated, with Firth’s bias corrected penalized Cox regression method [39] applied for subgroup analyses with a small number of events. A test with a *P*-value < 0.05 was considered statistically significant. All tests were two-sided. P-values were not adjusted for multiple testing. All statistical analyses were performed using SAS 9.1 software (SAS Institute, Inc.). Survival curves were plotted using R statistical software, version 2.15.0 (http://www.r-project.org/).

## Results and discussion

### Clinicopathological characteristics

The patient and tumor characteristics of the subgroup of 403 patients for which Mena^calc^ was obtained and the remaining subset of the TMA cohort (n = 888) are summarized in Table 1. Compared to the remaining cohort (n = 483), patients included in the Mena^calc^ analysis were of younger age, more likely to be pre-menopausal and more likely to have larger tumors.

**Table 1:** Association between clinical markers and Mena^calc^ expression availability in the TMA cohort (n = 888)

Characteristic		Mena^calc^ score available		Mena^calc^ score unavailable		*P*-value^d^
		(*n* = 403^a^)		(*n* = 483)		
		Number	%	Number	%	
Menopausal status						
	pre	151	37.5	141	29.2	0.0330
	peri	19	4.7	25	5.2	
	post	233	57.8	317	65.6	
Tumor Size						
	<0.5 cm	5	1.2	11	2.3	0.0198
	0.5 to < 1.0 cm	41	10.2	81	16.8	
	1.0 to < 2.0 cm	174	43.2	199	41.2	
	2 to 5 cm	164	40.7	179	37.0	
	>5 cm	19	4.7	13	2.7	
Estrogen receptor						
	Positive	239	59.3	307	63.6	0.2806
	Negative/Equivocal	104	25.8	103	21.3	
	ND^b^	60	14.9	73	15.1	
Progesterone receptor						
	Positive	215	53.3	280	58.0	0.2699
	Negative/Equivocal	128	31.8	130	26.9	
	ND^b^	60	14.9	73	15.1	
Histological grade						
	1^c^	99	24.6	174	36.0	<0.0001
	2	147	36.4	152	31.5	
	3	132	32.8	100	20.7	
	ND^b^	25	6.2	57	11.8	
Adjuvant treatment						
	Hormonal	169	41.9	205	42.4	0.0571
	Chemotherapy	78	19.4	64	13.3	
	Both	14	3.5	14	2.9	
	None	142	35.2	200	41.4	
Lymphatic Invasion						
	Yes	70	17.4	47	9.7	0.0008
	No	332	82.6	436	90.3	
	Missing	1^e^		0		
Age group						
	<50 yrs	157	39.0	149	30.9	0.0115
	≥50 yrs	246	61.0	334	69.1	

^a^without patients with most baseline unavailable data

^b^Unknown, not done or missing

^c^Includes mucinous, lobular and tubular subtypes

^d^Chi-square test

(^e^without missing category)

The patient and tumor characteristics of the Mena^calc^ high (at or above the median) and low (below the median) groups are summarized in Table 2. Tumors with high Mena^calc^ values were more likely to be higher grade and to have lymphatic invasion. These patients were also more likely to have received hormonal therapy and/or chemotherapy. 58 deaths were observed during follow-up. Excluding deaths and a small number of drop-outs, minimum and median follow-up times were 56.1 and 96.5 months respectively. Of the 54 recurrences observed, 15 patients presented with bone metastases alone, 14 patients presented with chest wall or regional lymph node involvement (either axillary or supraclavicular), 8 patients presented with lung metastases alone, 2 patients presented with liver metastases alone, 1 patient presented with skin metastases alone and 1 patient presented with a solitary neck muscle deposit. 8 patients presented with bone and liver metastases and 3 patients presented with bone and lung metastases. 2 patients presented with widespread multi-organ involvement (bone, liver and lung). The average time to recurrence was 41.8 months (s.d. = 23 months). A table outlining the average time and range of time to recurrence for each metastases subgroup is included as Additional file 1: Table S1.

**Table 2:** Association between clinical markers and Mena^calc^ expression (n = 403)

Characteristic		Mena^calc^ low		Mena^calc^ high		*P*-value^c^
		(*n* = 202)		(*n* = 201)		
		Number	%	Number	%	
Death						
	Yes	22	10.9	36	17.9	0.0447
	No	180	89.1	165	82.1	
Menopausal status						
	pre	72	35.6	79	39.3	0.6969
	peri	9	4.5	10	5.0	
	post	121	59.9	112	55.7	
Tumor Size						
	<0.5 cm	2	1.0	3	1.5	0.8845
	0.5 to < 1.0 cm	21	10.4	20	10.0	
	1.0 to < 2.0 cm	91	45.0	83	41.2	
	2 to 5 cm	80	39.6	84	41.8	
	>5 cm	8	4.0	11	5.5	
Estrogen receptor						
	Positive	124	61.4	115	57.2	0.3701
	Negative/Equivocal	46	22.8	58	28.9	0.1926^d^
	ND^a^	32	15.8	28	13.9	
Progesterone receptor						
	Positive	112	55.5	103	51.3	0.4136
	Negative/Equivocal	58	28.7	70	34.8	0.2245^d^
	ND^a^	32	15.8	28	13.9	
Histological grade						
	1^b^	58	28.7	41	20.4	0.2389
	2	71	35.2	76	37.8	
	3	60	29.7	72	35.8	
	ND^a^	13	6.4	12	6.0	
Adjuvant treatment						
	Hormonal	76	37.6	93	46.3	0.0985
	Chemotherapy	37	18.3	41	20.4	
	Both	10	5.0	4	2.0	
	None	79	39.1	63	31.3	
Lymphatic Invasion						
	Yes	27	13.4	43	21.5	0.0315
	No	175	86.6	157	78.5	
	Missing	0		1		
Age group						
	<50 yrs	73	36.1	84	41.8	0.2447
	≥50 yrs	129	63.9	117	58.2	

^a^Unknown, not done or missing

^b^Includes mucinous, lobular and tubular subtypes

^c^By Chi-square test (without Missing category)

^d^Without ND group

### Association of Mena^calc^ with patient survival

#### Full group (n = 403)

**Fig. 1:**
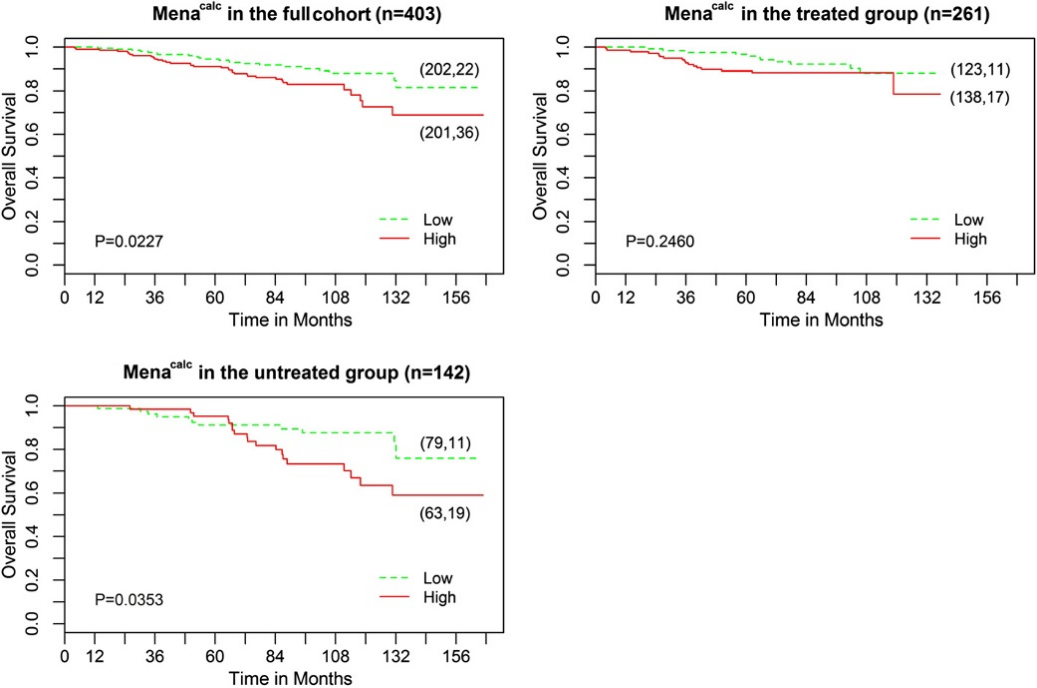
Kaplan-Meier analysis of Mena^calc^ in the ANN patient cohort (n = 403, top left), in a subset of patients who received chemotherapy and/or hormone therapy (n = 261, top right) and in a subset of the patient population that did not receive chemotherapy or hormone therapy (n = 142, bottom left). Mena^calc^ scores were categorized as Mena^calc^ high if they were at or above the median and Mena^calc^ low if they were below the median. In brackets is the total number of patients followed by the number of patient deaths for each group

Women in the Mena^calc^ low group had significantly better overall survival compared to women in the Mena^calc^ high group (Fig. 1: K-M survival curves; Log-Rank *P* = 0.0227). In univariate Cox regression analysis, when Mena^calc^ status was considered alone, there was a 1.84-fold (CI = (1.08, 3.14), *P* = 0.0248) higher risk of death in the Mena^calc^ high group (Table 3). The magnitude and significance of the Mena^calc^ high association with death persisted with adjustment for HER2 status, hormone receptor status and other clinicopathological tumor variables (HR = 2.18, CI = (1.19, 4.00), *P* = 0.0199) (Table 3).

**Table 3:** Results of Overall Survival Analysis by Cox Proportional Hazards Model for the full dataset

Prognostic Factor		Univariate (*n* = 403)		Multivariate (*n* = 360^c^)	
		HR (95 % CI)	P-value	HR (95 % CI)	*P*-value
Mena^calc^					
	High vs. Low	1.84 (1.08, 3.14)	0.0248	2.18 (1.19, 4.00)	0.0199
Her2^a^					
	Positive vs. Negative	1.71 (0.77, 3.79)	0.1855	1.43 (0.58, 3.53)	0.4341
Tumor Size					
	≥2 cm vs. <2 cm	1.40 (0.83, 2.35)	0.2024	1.45 (0.80, 2.66)	0.2234
ER^a^					
	Negative vs. Positive	1.18 (0.65, 2.13)	0.5858	1.09 (0.57, 2.08)	0.7952
PR^a^					
	Positive vs. Negative	2.36 (1.32, 4.25)	0.0040	NA^b^	NA^b^
Histological grade					
	Grade 2-3 vs. Grade 1	1.62 (0.82, 3.22)	0.1682	1.27 (0.59, 2.72)	0.5450
	ND vs. Grade 1	1.06 (0.33, 3.42)	0.9264	0.75 (0.22, 2.49)	0.6354
Lymphatic invasion					
	Present vs. Absent	1.78 (0.97, 3.26)	0.0614	1.67 (0.85, 3.30)	0.1359
Treatment					
	Yes vs. No	0.60 (0.35, 1.00)	0.0514	0.54 (0.29, 0.99)	0.0454
Age, years					
	≥50 vs. <50	1.90 (1.06, 3.43)	0.0319	1.93 (1.02, 3.65)	0.0425

^a^IHC marker

^b^PR was not included as ER and PR are correlated

^c^Tumors excluded if missing data for either Her2 or ER

A similar association to the full group findings was observed when the analysis was restricted to patients who had received no systemic adjuvant treatment (Log-Rank *P* = 0.0353, Fig. 1). When Mena^calc^ status was considered alone, there was a 2.14-fold (CI = (1.05, 4.58), *P* = 0.0445) higher risk of death in the Mena^calc^ high group (Table 4). The magnitude and the significance of the association of high Mena^calc^ with death persisted with adjustment for the same variables as for the full group (HR = 3.80, CI = (1.58, 9.97), *P* = 0.0052) (Table 4). An association was not detected in the treated group (Fig. 1), but a test comparing the Mena^calc^ association in the treated versus the untreated group (MV HR = 1.38 versus HR = 3.37) was equivocal due to low power to detect interaction (ratio of treated versus untreated MV HRs = 0.41, CI = (0.12, 1.32), *P* = 0.1500) (data not shown).

**Table 4:** Results of Overall Survival Analysis by Cox Proportional Hazards Model for the untreated subgroup

Prognostic Factor		Univariate (*n* = 142)		Multivariate (*n* = 129^c^)	
		HR (95 % CI)	*P*-value	HR (95 % CI)	*P*-value
Mena^calc^					
	High vs. Low	2.14 (1.05, 4.58)	0.0445	3.80 (1.58, 9.97)	0.0052
Her2^a^					
	Positive vs. Negative	1.59 (0.43, 4.31)	0.4234	1.35 (0.34, 4.02)	0.6376
Tumor Size					
	≥2 cm vs. <2 cm	2.05 (1.01, 4.22)	0.0508	1.41 (0.62, 3.23)	0.4178
ER^a^					
	Negative vs. Positive	1.62 (0.68, 3.51)	0.2504	0.99 (0.36, 2.51)	0.9863
PR*					
	Positive vs. Negative	2.84 (1.27, 7.05)	0.0173	NA^b^	NA^b^
Histological grade					
	Grade 2-3 vs. Grade 1	2.61 (1.09, 7.43)	0.0505	1.85 (0.67, 5.84)	0.2719
	ND vs. Grade 1	1.46 (0.39, 5.23)	0.5667	1.34 (0.33, 5.03)	0.6763
Lymphatic invasion					
	Present vs. Absent	2.11 (0.81, 4.75)	0.0993	1.63 (0.54, 4.16)	0.3537
Age, years					
	≥50 vs. <50	2.41 (1.03, 6.77)	0.0666	3.03 (1.17, 9.11)	0.0348

^a^IHC marker

^b^PR was not included as ER and PR are correlated

^c^Tumors excluded if missing data for either Her2 or ER

#### Molecular subtypes (n = 233)

**Fig. 2:**
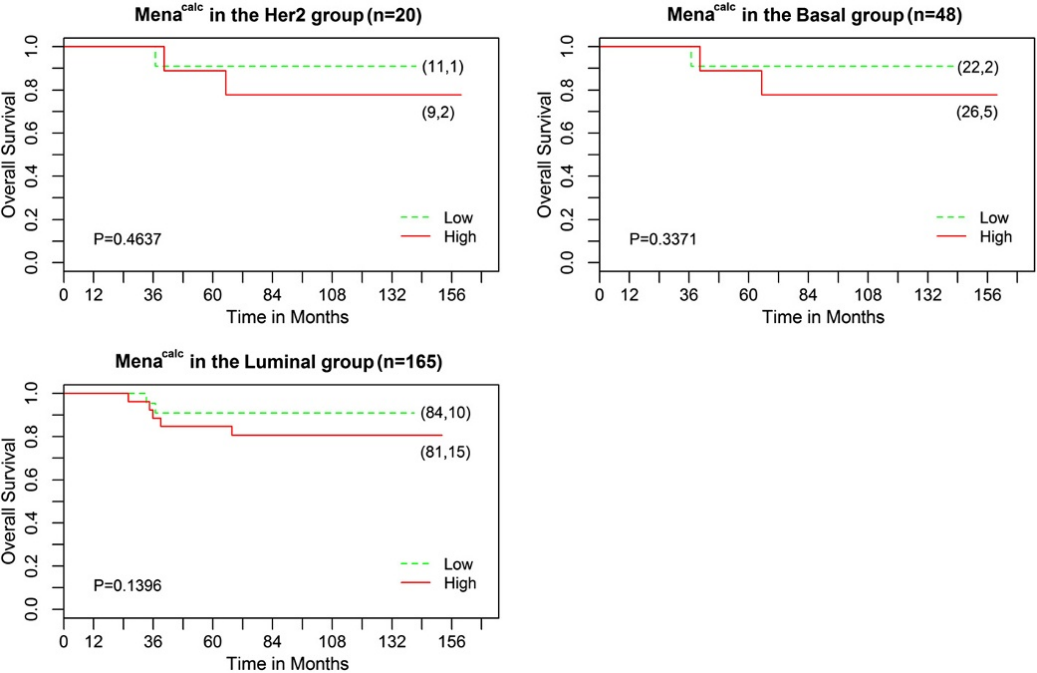
Kaplan-Meier analysis of Mena^calc^ for ANN tumors subclassified by immunohistochemical subtype: HER2 amplified (n = 20, top left), basal (n = 48, top right) and luminal (n = 165, bottom left). In brackets is the total number of patients followed by the number of patient deaths for each group

When the tumors were subdivided into immunohistochemical subtypes, 8.5 % were classified as HER2, 20.5 % were classified as basal, and 70.5 % were classified as luminal. Fig. 2 shows K-M survival curves for the association between the Mena^calc^ status (high vs. low) and survival in the three main subtype groups: HER2 (n = 20), basal (n = 48) and luminal (n = 165). Although the subtype tests of association did not attain nominal 5 % significance, the plots show the same trend of high Mena^calc^ association with worse survival.

## Conclusions

The findings of this study suggest that Mena^calc^ is prognostic for ANN breast cancer. While high Mena^calc^ values were correlated with poor prognostic features (i.e., high tumor grade, lymphatic invasion), they were also associated with decreased overall survival in our cohort of 403 ANN breast cancer patients, independent of standard prognostic variables. These results complement our previous findings in two independent cohorts of breast cancer patients which indicated that relatively high Mena^calc^ values were associated with increased risk of death from breast cancer [29]. However, in the previous study, where the number of ANN patients was considerably less than in the present study, Mena^calc^ was not associated with risk of death from breast cancer in the ANN subgroup.

While Mena^calc^ may have clinical utility, there were two main limitations which may impact interpretation of our results. First of all, due to limited tumor material, the cohort was skewed to consist primarily of young women with larger tumors and a luminal immunohistochemical profile. Although Mena^calc^ may be able to subdivide these patients into low and high risk of recurrence groups, these patients are already considered to be at high risk of negative outcome and are usually managed aggressively. Second of all, this study did not have a validation cohort which would have helped to better assess the prognostic capabilities of Mena^calc^.

Mena^calc^ has been shown to be an independent negative prognostic marker in three breast cancer patient cohorts, including this ANN cohort. Taken together, these findings strongly suggest that Mena^calc^ should be investigated further as a potential clinical tool. Future studies could explore the predictive capabilities of Mena^calc^ in older ANN patients with a lower risk of recurrence (i.e., smaller tumor size). Also, investigating the prognostic value of Mena^calc^ in a cohort with larger proportions of the other molecular subtypes (i.e., basal, HER2) may uncover an association with specific biological/ clinical behavior. Finally, while findings on TMA specimens are promising, future work could compare the performance of Mena^calc^ on TMAs to that seen on whole slide specimens as a step towards use in clinical practice.

## Additional file

Additional file 1: Table S1. Time to disease recurrence for the sites of metastasis in the ANN cohort.

## Abbreviations

ANN: Axillary node-negative
AQUA: Automated quantitative analysis
CK5: Cytokeratin 5
CI: Confidence interval
EGF: Epidermal growth factor
EGFR: Epidermal growth factor receptor
Ena: Enabled
ER: Estrogen receptor
HER2: Human epidermal growth factor receptor 2
HR: Hazard ratio
IHC: Immunohistochemical
K-M: Kaplan Meier
LVI: Lymphatic invasion
MV: Multivariate
OS: Overall survival
PH: Proportional hazards
PgR: Progesterone receptor
RT-PCR: Real-time polymerase chain reaction
TMA: Tissue microarray
TMEM: Tumor microenvironment of metastasis
UV: Univariate
VASP: Vasodilator-stimulated phosphoprotein
VEGF: Vascular endothelial growth factor

## References

[1] Vaz-Luis I, Ottesen RA, Hughes ME, Mamet R, Burstein HJ, Edge SB (2014). Outcomes by tumor subtype and treatment pattern in women with small, node-negative breast cancer: a multi-institutional study. J Clin Oncol.

[2] Fehrenbacher L, Capra AM, Quesenberry CP, Fulton R, Shiraz P, Habel LA (2014). Distant invasive breast cancer recurrence risk in human epidermal growth factor receptor 2-positive T1a and T1b node-negative localized breast cancer diagnosed from 2000 to 2006: a cohort from an integrated health care delivery system. J Clin Oncol.

[3] Gonzalez-Angulo AM, Litton JK, Broglio KR, Meric-Bernstam F, Rakkhit R, Cardoso F (2009). High risk of recurrence for patients with breast cancer who have human epidermal growth factor receptor 2-positive, node-negative tumors 1 cm or smaller. J Clin Oncol.

[4] Sorlie T, Perou CM, Tibshirani R, Aas T, Geisler S, Johnsen H (2001). Gene expression patterns of breast carcinomas distinguish tumor subclasses with clinical implications. Proc Natl Acad Sci USA.

[5] Veer LJ V 't, Dai H, van de Vijver MJ, He YD, Hart AA, Mao M (2002). Gene expression profiling predicts clinical outcome of breast cancer. Nature.

[6] Blows FM, Driver KE, Schmidt MK, Broeks A, van Leeuwen FE, Wesseling J (2010). Subtyping of breast cancer by immunohistochemistry to investigate a relationship between subtype and short and long term survival: a collaborative analysis of data for 10,159 cases from 12 studies. PLoS Med.

[7] Gertler FB, Niebuhr K, Reinhard M, Wehland J, Soriano P (1996). Mena, a relative of VASP and Drosophila Enabled, is implicated in the control of microfilament dynamics. Cell.

[8] Barzik M, Kotova TI, Higgs HN, Hazelwood L, Hanein D, Gertler FB (2005). Ena/VASP proteins enhance actin polymerization in the presence of barbed end capping proteins. J Biol Chem.

[9] Di Modugno F, Bronzi G, Scanlan MJ, Del Bello D, Cascioli S, Venturo I (2004). Human Mena protein, a serex-defined antigen overexpressed in breast cancer eliciting both humoral and CD8+ T-cell immune response. Int J Cancer.

[10] Di Modugno F, Mottolese M, Di Benedetto A, Conidi A, Novelli F, Perracchio L (2006). The cytoskeleton regulatory protein hMena (ENAH) is overexpressed in human benign breast lesions with high risk of transformation and human epidermal growth factor receptor-2-positive/hormonal receptor-negative tumors. Clin Cancer Res.

[11] Goswami S, Philippar U, Sun D, Patsialou A, Avraham J, Wang W (2009). Identification of invasion specific splice variants of the cytoskeletal protein Mena present in mammary tumor cells during invasion in vivo. Clin Exp Metastasis.

[12] Gertler F, Condeelis J (2011). Metastasis: tumor cells becoming MENAcing. Trends Cell Biol.

[13] Wang W, Goswami S, Lapidus K, Wells AL, Wyckoff JB, Sahai E (2004). Identification and testing of a gene expression signature of invasive carcinoma cells within primary mammary tumors. Cancer Res.

[14] Wang W, Wyckoff JB, Goswami S, Wang Y, Sidani M, Segall JE (2007). Coordinated regulation of pathways for enhanced cell motility and chemotaxis is conserved in rat and mouse mammary tumors. Cancer Res.

[15] Patsialou A, Wang Y, Lin J, Whitney K, Goswami S, Kenny PA (2012). Selective gene-expression profiling of migratory tumor cells in vivo predicts clinical outcome in breast cancer patients. Breast Cancer Res.

[16] Patsialou A, Bravo-Cordero JJ, Wang Y, Entenberg D, Liu H, Clarke M (2013). Intravital multiphoton imaging reveals multicellular streaming as a crucial component of in vivo cell migration in human breast tumors. Intravital.

[17] Philippar U, Roussos ET, Oser M, Yamaguchi H, Kim HD, Giampieri S (2008). A Mena invasion isoform potentiates EGF-induced carcinoma cell invasion and metastasis. Dev Cell.

[18] Roussos ET, Balsamo M, Alford SK, Wyckoff JB, Gligorijevic B, Wang Y (2011). Mena invasive (MenaINV) promotes multicellular streaming motility and transendothelial migration in a mouse model of breast cancer. J Cell Sci.

[19] Roussos ET, Goswami S, Balsamo M, Wang Y, Stobezki R, Adler E (2011). Mena invasive (Mena(INV)) and Mena11a isoforms play distinct roles in breast cancer cell cohesion and association with TMEM. Clin Exp Metastasis.

[20] Roussos ET, Wang Y, Wyckoff JB, Sellers RS, Wang W, Li J (2010). Mena deficiency delays tumor progression and decreases metastasis in polyoma middle-T transgenic mouse mammary tumors. Breast Cancer Res.

[21] Wyckoff JB, Wang Y, Lin EY, Li JF, Goswami S, Stanley ER (2007). Direct visualization of macrophage-assisted tumor cell intravasation in mammary tumors. Cancer Res.

[22] Harney AS, Arwert EN, Entenberg D, Wang Y, Guo P, Qian B-Z et al. Real-time imaging of the tumor microenvironment reveals local, transient vascular permeability and tumor cell intravasation stimulated by macrophage-derived VEGF. Cancer Discovery. 2015; in press. 10.1158/2159-8290.CD-15-0012 PMC4560669 26269515

[23] Wyckoff J, Wang W, Lin EY, Wang Y, Pixley F, Stanley ER (2004). A paracrine loop between tumor cells and macrophages is required for tumor cell migration in mammary tumors. Cancer Res.

[24] Roussos ET, Condeelis JS, Patsialou A (2011). Chemotaxis in cancer. Nat Rev Cancer.

[25] Shapiro IM, Cheng AW, Flytzanis NC, Balsamo M, Condeelis JS, Oktay MH (2011). An EMT-driven alternative splicing program occurs in human breast cancer and modulates cellular phenotype. PLoS Gen.

[26] Pignatelli J, Goswami S, Jones JG, Rohan TE, Pieri E, Chen X (2014). Invasive breast carcinoma cells from patients exhibit MenaINV- and macrophage-dependent transendothelial migration. Sci Signal.

[27] Robinson BD, Sica GL, Liu YF, Rohan TE, Gertler FB, Condeelis JS (2009). Tumor microenvironment of metastasis in human breast carcinoma: a potential prognostic marker linked to hematogenous dissemination. Clin Cancer Res.

[28] Rohan TE, Xue X, Lin HM, D'Alfonso TM, Ginter PS, Oktay MH et al. Tumor microenvironment of metastasis and risk of distant metastasis of breast cancer. J Natl Cancer Inst. 2014;106(8). doi:10.1093/jnci/dju136. 10.1093/jnci/dju136 PMC4133559 24895374

[29] Agarwal S, Gertler FB, Balsamo M, Condeelis JS, Camp RL, Xue X (2012). Quantitative assessment of invasive mena isoforms (Menacalc) as an independent prognostic marker in breast cancer. Breast Cancer Res.

[30] Bull SB, Ozcelik H, Pinnaduwage D, Blackstein ME, Sutherland DA, Pritchard KI (2004). The combination of p53 mutation and neu/erbB-2 amplification is associated with poor survival in node-negative breast cancer. J Clin Oncol.

[31] Andrulis IL, Bull SB, Blackstein ME, Sutherland D, Mak C, Sidlofsky S (1998). neu/erbB-2 amplification identifies a poor-prognosis group of women with node-negative breast cancer. Toronto Breast Cancer Study Group. J Clin Oncol.

[32] McShane LM, Altman DG, Sauerbrei W, Taube SE, Gion M, Clark GM (2005). REporting recommendations for tumour MARKer prognostic studies (REMARK). Br J Cancer.

[33] Welsh AW, Moeder CB, Kumar S, Gershkovich P, Alarid ET, Harigopal M (2011). Standardization of estrogen receptor measurement in breast cancer suggests false-negative results are a function of threshold intensity rather than percentage of positive cells. J Clin Oncol.

[34] Camp RL, Chung GG, Rimm DL (2002). Automated subcellular localization and quantification of protein expression in tissue microarrays. Nat Med.

[35] Moeder CB, Giltnane JM, Moulis SP, Rimm DL (2009). Quantitative, fluorescence-based in-situ assessment of protein expression. Methods Mol Biol..

[36] Kornegoor R, Verschuur-Maes AH, Buerger H, Hogenes MC, de Bruin PC, Oudejans JJ (2012). Molecular subtyping of male breast cancer by immunohistochemistry. Mod Pathol.

[37] Livasy CA, Karaca G, Nanda R, Tretiakova MS, Olopade OI, Moore DT (2006). Phenotypic evaluation of the basal-like subtype of invasive breast carcinoma. Mod Pathol.

[38] Voduc KD, Cheang MC, Tyldesley S, Gelmon K, Nielsen TO, Kennecke H (2010). Breast cancer subtypes and the risk of local and regional relapse. J Clin Oncol.

[39] Heinze G, Schemper M (2001). A solution to the problem of monotone likelihood in Cox regression. Biometrics.

